# Rosmarinic Acid Delays Tomato Fruit Ripening by Regulating Ripening-Associated Traits

**DOI:** 10.3390/antiox10111821

**Published:** 2021-11-17

**Authors:** Changan Zhu, Shaofang Wu, Ting Sun, Zhiwen Zhou, Zhangjian Hu, Jingquan Yu

**Affiliations:** 1 Department of Horticulture, Zhejiang University, Hangzhou 310058, China; 12016055@zju.edu.cn (C.Z.); 11816046@zju.edu.cn (S.W.); 11916066@zju.edu.cn (T.S.); 21916058@zju.edu.cn (Z.Z.); jqyu@zju.edu.cn (J.Y.); 2 Shandong (Linyi) Institute of Modern Agriculture, Zhejiang University, Linyi 276000, China

**Keywords:** rosmarinic acid, fruit ripening, tomato, flavor quality, ethylene, antioxidant system

## Abstract

Fruits are excellent sources of essential vitamins and health-boosting minerals. Recently, regulation of fruit ripening by both internal and external cues for the improvement of fruit quality and shelf life has received considerable attention. Rosmarinic acid (RA) is a kind of natural plant-derived polyphenol, widely used in the drug therapy and food industry due to its distinct physiological functions. However, the role of RA in plant growth and development, especially at the postharvest period of fruits, remains largely unknown. Here, we demonstrated that postharvest RA treatment delayed the ripening in tomato fruits. Exogenous application of RA decreased ripening-associated ethylene production and inhibited the fruit color change from green to red based on the decline in lycopene accumulation. We also found that the degradation of sucrose and malic acid during ripening was significantly suppressed in RA-treated tomato fruits. The results of metabolite profiling showed that RA application promoted the accumulation of multiple amino acids in tomato fruits, such as aspartic acid, serine, tyrosine, and proline. Meanwhile, RA application also strengthened the antioxidant system by increasing both the activity of antioxidant enzymes and the contents of reduced forms of antioxidants. These findings not only unveiled a novel function of RA in fruit ripening, but also indicated an attractive strategy to manage and improve shelf life, flavor, and sensory evolution of tomato fruits.

## 1. Introduction

Tomato is regarded as an important horticultural crop worldwide. As a rich source of carbohydrates, vitamins, dietary fiber, and antioxidants, fleshy tomato fruits provide humans with numerous health benefits [1]. Fruit ripening is a well-orchestrated and important development process in the life cycle of tomatoes. Traditionally, fruit ripening has been classified into two types, such as the climacteric type and the nonclimacteric type. Tomatoes belong to typical climacteric fruits, showing a characteristic burst in respiration rates and ethylene production at or just before the onset of ripening coinciding with massive transcriptional changes [2]. Understanding the underlying mechanisms of fruit ripening is extremely essential for enhancing our scientific knowledge to improve fruit quality, nutritional values, and shelf life towards reducing fruit waste and economic losses in the tomato industry.

Generally, tomato fruit ripening is characterized by softening, color changes, and accumulations of flavor and aromatic compounds [3]. Fruit softening is regarded as a consequence of cell wall disassembly and the changes in cellular turgor [4]. The fruit color change from green to red is associated with the degradation of chlorophylls and the simultaneous accumulation of carotenoids such as lycopene, β-carotene, and lutein. Among these carotenoids, lycopene is considered a major component that contributes to the formation of red color in tomato fruits [5]. Meanwhile, fruit ripening triggers a substantial accumulation of carbohydrates, that provide energy for fruit developmental changes and also trigger ripening-associated sugar signal pathways. The major form of carbohydrates imported into fruits from the photosynthetic organs is sucrose, which is also synthesized in the cytosol, vacuole, and apoplast of fruit cells [6]. During ripening, sucrose can be irreversibly hydrolyzed by invertase into glucose and fructose, or cleaved by sucrose synthase into fructose and uridine diphosphate-(UDP-) glucose in the presence of UDP [7]. These distinct sugar metabolic processes change the content and component of sugars, and remodel fruit flavor and total soluble solids. Besides, carbohydrate accumulation, dramatic changes in free amino acids, organic acids, and volatiles occur during tomato fruit ripening [8].

Ethylene production is regarded as a critical indicator of fruit ripening, and also is the major cue controlling most aspects of ripening in climacteric fruits. To avoid the postharvest losses caused by the early onset of ripening, it is a good strategy to either block or inhibit ethylene synthesis. The key enzymes involved in ethylene synthesis include 1-aminocyclopropane-1-carboxylic acid (ACC) synthase (ACS) and ACC oxidase ACO. ACS contributes to ACC production from S-adenosylmethionine (SAM), and ACO is responsible for the conversion of ACC into ethylene. Genetic mutations in ethylene biosynthesis genes affect tomato fruit ripening [3]. Meanwhile, exogenous application of the potent ethylene perception inhibitor 1-methylcyclopropene (1-MCP) can remarkably delay fruit ripening [9]. However, the inhibition of fruit ripening by the above-mentioned approaches also results in an accompanying loss in flavor quality of tomato fruits.

Except for the ethylene burst, increased respiration rates during tomato fruit ripening are associated with oxidative processes, producing reactive oxygen species (ROS), such as ephemeral singlet oxygen, superoxide radical (O_2_^•−^), and hydrogen peroxide (H_2_O_2_). At low and moderate concentrations, ROS can function as a second messenger to transduce signaling cascades that mediate many plant physiological responses during fruit ripening [10]. However, high ROS accumulation promoted by water loss, ethylene production, and respiration induces lipid peroxidation in cells during postharvest storage, exacerbating oxidative stress and protein damages. Therefore, plants develop a complex antioxidative defense system, which includes enzymatic and non-enzymatic components. The enzymatic components, such as superoxide dismutase (SOD), catalase (CAT), and the enzymes involved in the ascorbate-glutathione cycle, including ascorbate peroxidase (APX), glutathione reductase (GR), dehydroascorbate reductase (DHAR), and monodehydroascorbate reductase (MDHAR), constitute the first layer of defense against oxidative stress [11]. Meanwhile, non-enzymatic antioxidants such as ascorbate, glutathione, carotenoids, tocopherols and polyphenols work in concert with antioxidant enzymes to maintain a steady state of redox homeostasis [12]. Among the non-enzymatic antioxidants, polyphenols are the most abundant antioxidant in plant fruits, and can directly capture oxidative free radicals [13].

Rosmarinic acid (RA), named after rosemary (*Rosmarinus officinaliss* L.) plants, is a classical type of polyphenol, widely found in various plant species, ranging from the hornworts (Anthocerotaceae, one of the earliest land plant families) to eudicotyledonous plants (many species from the families Lamiaceae and Boraginaceae) [14]. Biosynthesis of RA initiates from the aromatic amino acids L-phenylalanine and L-tyrosine, which is transformed to the intermediary precursors 4-coumaroyl-CoA by the general phenylpropanoid pathway and 4-hydroxyphenyllactic acid, respectively. Then, the two intermediary precursors are coupled to form 4-coumaroyl-4′-hydroxyphenyllactic acid (4C-pHPL) by rosmarinic acid synthase, and the hydroxyl groups of RA are eventually added by cytochrome P450-relied monooxygenase reactions [14]. However, whether RA can be biosynthesized in tomato has not been characterized yet. RA has a number of potentially beneficial pharmacological functions, and it exhibits antiallergic, anti-inflammatory, anticarcinogenic, antimicrobial, and neuroprotective properties [15]. In addition, RA can scavenge free radicals, chelate pro-oxidant ions, and prevent lipid peroxidation. Therefore, RA is regarded as a natural antioxidant with a variety of valuable applications in the food industry [16]. However, the role of RA in fruit ripening remains poorly understood, which limits its application in fruit postharvest storage.

In this study, we explored the effect of RA on tomato fruit ripening, and revealed that RA delayed tomato fruit ripening and regulated distinct biochemical routes associated with fruit sensory and flavor traits. Our results have potential significance in providing a novel strategy to maintain nutritional quality and extend shelf life in tomato fruits.

## 2. Materials and Methods

### 2.1. Plant Materials and Treatments

Tomato (*Solanum lycopersicum* L. cv. Zheyingfen 1) seeds were obtained from Zhejiang Academy of Agricultural Sciences. Tomato plants were cultivated in a greenhouse as described in a previous study [17]. Tomato fruits with uniform size were harvested at the mature green stage (35 d post anthesis) for the next experiments.

A powdered form of RA (Cat. R109805, Aladdin, China) was dissolved in distilled water (dH_2_O) to prepare the graded levels of working solutions with the concentration of 0.25, 0.5, and 0.75 mM. Harvested tomato fruits were classified into four groups randomly, and then soaked in different concentration of RA working solutions, or dH_2_O for 30 min, respectively. Then, partially air-dried fruits were collected immediately as the samples of 0 d, and the rest fruits were stored in identical condition (25 °C, 85% relative humanity, and 16 h/8 h of day/night photoperiod), and sampled at 4, 8, 12, and 16 d after the soaking treatment. Three biological replicates of at least three fruits each were ground into fine powder in liquid nitrogen, and stored at −80 °C until further analysis.

### 2.2. Fruit Firmness Measurement

The firmness of tomato fruits pericarp was tested by the TA-XT2i texture analyzer (Stable Micro Systems Ltd., Godalming, UK) with a black plastic probe of 5.0 mm in diameter. The penetration depth was 10 mm, and the penetration speed was set at 1 mm·s^−1^. Newton (N) is represented as the unit of force. Nine biological replicates were used for each group.

### 2.3. Ethylene Production Quantification

Ethylene production was determined based on the previous study [18]. Briefly, four tomato fruits were sealed into a 500 mL gas-tight jar at 25 °C for 1 h. Then, a 1 mL gas sample, from the headspace, was withdrawn using a syringe and injected into a gas chromatograph (6890N GC system; Agilent, Folsom, CA, USA) equipped with a flame ionization detector. In addition, the weight of tomato fruits was recorded for calculating the ethylene production on the basis of per gram of fresh weight (FW).

### 2.4. Carotenoid Content Measurement

Briefly, 0.3 g fruit powder was vortexed with 700 μL chloroform, 350 μL double distilled H_2_O (ddH_2_O) and 350 μL methanol in a 2 mL tube. The chloroform phase was collected by centrifugation at 10,000× *g* for 10 min at 4 °C. The chloroform extraction was repeated three times. After being dried under flowing nitrogen gas, the residue was dissolved in 150 μL ethyl acetate (HPLC grade), and 100 μL was transferred into a new tube for HPLC analysis. Carotenoid content was analyzed by a Waters Liquid Chromatography System (e2695) equipped with a 20 × 4.6 mm inner diameter (i.d)., YMC C30 guard column and a 250 × 4.6 mm i.d., 5 μm, YMC reverse-phase C30 column, according to the method described previously [18].

### 2.5. Sugar and Organic Acid Measurement

A total of 0.1 g tomato fruit powder was weighed into a 10 mL tube, and then 1mL double-distilled water (ddH_2_O) was added, followed by vortexing. The homogenates were incubated at 80 °C for 30 min. After the centrifugation at 12,000× *g* for 10 min, the supernatant was added with the same volume ddH_2_O for another incubation at 80 °C for 30 min. After the centrifugation at 12,000× *g* for 10 min, a part of supernatant (2 μL for sucrose, malic acid, and citric acid; 100 μL for glucose and fructose) was fulfilled with 80% acetonitrile (HPLC grade) to 1 mL, among which 200 μL solution was transferred to HPLC analysis tubes.

Sugars and organic acids were analyzed by the HPLC (Waters Alliance 2695 system, Waters Corporation, Milford, MA, USA) with a refractive index detector RI-1530 (Jasco Corp., Tokyo, Japan) according to [19]. Acetonitrile: water (80:20, *v/v*) with a flow rate of 1.0 mL min^−1^ was used as the mobile phase. Standard samples of sucrose (Solarbio, Beijing, China), glucose (Solarbio, Beijing, China), fructose (Solarbio, Beijing, China), malic acid (Solarbio, Beijing, China) and citric acid (Solarbio, Beijing, China) were measured to calculate the individual sugar or organic acid in fruit samples.

### 2.6. Total RNA Extraction and qRT-PCR Analyses

Total RNA was extracted from the tomato fruit powder using the RNA extraction kit (Omega Bio Tek, Norcross, GA, USA) following the manufacturer’s instructions. 0.5 μg of total RNA was reverse transcribed into cDNA using a HiScript II Q RT SuperMix for qPCR Kit (Vazyme Biotech, Nanjing, China). The qRT-PCR experiments were conducted on the Light Cycler^®^ 480 II Real-Time PCR Detection System (Roche, Swiss). The tomato housekeeping gene *ACTIN* (Solyc03g078400) was used as internal control to calculate the relative expression of target genes. PCR was performed with 3 min at 95 °C, followed by 45 cycles of 30 sec at 95 °C, 30 sec at 58 °C, and 1 min at 72 °C. Sequences of primers are listed in Table S1.

### 2.7. Measurements of Antioxidant Contents and Enzyme Activities

For the measurement of antioxidant enzyme activity, 0.3 g tomato fruit powder was mixed with 3 mL ice-cold 50 mM phosphate buffer (pH 7.8, 25 mM HEPES, 0.2 mM EDTA, 2 mM AsA, and 2% polyvinylpolypyrrolidone (*w/v*)). After centrifugation (12,000× *g* for 20 min at 4 °C), the supernatants were collected for the subsequent enzyme activity assay using SHIMADZU UV-2410PC spectrophotometer (Shimadzu, Kyoto, Japan). The activity of APX, CAT, GR, and SOD were determined following the previously described method [20].

For the measurement of the antioxidant content, 0.1 g tomato fruit powder was extracted into 1 mL 0.2 M HCl. After centrifugation (14,000× *g* for 10 min at 4 °C), 0.5 mL supernatant was mixed with 100 μL 0.2 M phosphate buffer (pH 5.6), and then 0.2 M NaOH was added to adjust pH 4~5. Then ascorbate and glutathione contents were measured using a 96 well UV plate and a microplate reader [21].

### 2.8. Metabolite Profiling

80-mg tomato fruit sample powder was extracted overnight with 1 mL cold extraction solvent methanol/acetonitrile/H_2_O (2:2:1, *v/v/v*) at 4 °C. After centrifugation at 14,000× *g* for 20 min at 4 °C, the supernatant was collected and flowed through a 96-well protein precipitation plate, and then the elution was collected and dried in a vacuum centrifuge at 4 °C. The samples were re-dissolved in 100 μL acetonitrile/water (1:1, *v/v*) solvent and transferred to LC vials for LC-MS analysis. Untargeted metabolomic analysis was carried out using a UHPLC (1290 Infinity LC, Agilent Technologies, Santa Clara, CA, USA) with a quadrupole time-of-flight mass spectrometry (Sciex TripleTOF 6600, Framingham, MA, USA). Details of the instrumentation and data analysis were described previously [22]. Briefly, for LC separation, samples were analyzed using a 2.1 × 100 mm ACQUIY UPLC BEH 1.7 µm column (waters, Ireland). The mobile phase contained A = 25 mM ammonium acetate and 25 mM ammonium hydroxide in water and B = acetonitrile. The gradient was 85% B for 1 min and was linearly reduced to 65% in 11 min, and then was reduced to 40% in 0.1 min and kept for 4 min, and then increased to 85% in 0.1 min, with a 5 min re-equilibration period employed. The mass spectrometer was operated in both negative ion and positive ionizations mode. The ESI source conditions were set as follows: scan range *m/z* 60–1000, Ion Source Gas1 (Gas1) as 60, Ion Source Gas2 (Gas2) as 60, curtain gas (CUR) as 30, source temperature: 600 °C, IonSpray Voltage Floating (ISVF) ± 5500 V. The raw MS data (wiff.scan files) were converted to MzXML (an open data format for storage and exchange of mass spectroscopy data) files using ProteoWizard MSConvert and processed using XCMS Online (La Jolla, CA, USA) for feature detection, retention time correction and alignment.

### 2.9. Statistical Analysis

Data are expressed as mean ± SD and were analyzed using GraphPad InStat software 8.3.0 for Windows (GraphPad Software Inc., La Jolla, CA, USA) and R software. Student’s *t*-test was applied for comparison between the control and RA treatment group. Differences are considered as statistically significant at the value of * *p* < 0.05, ** *p* < 0.01.

## 3. Results

### 3.1. Effect of RA Treatment on the Sensory Quality of Tomato Fruits

**Figure 1:**
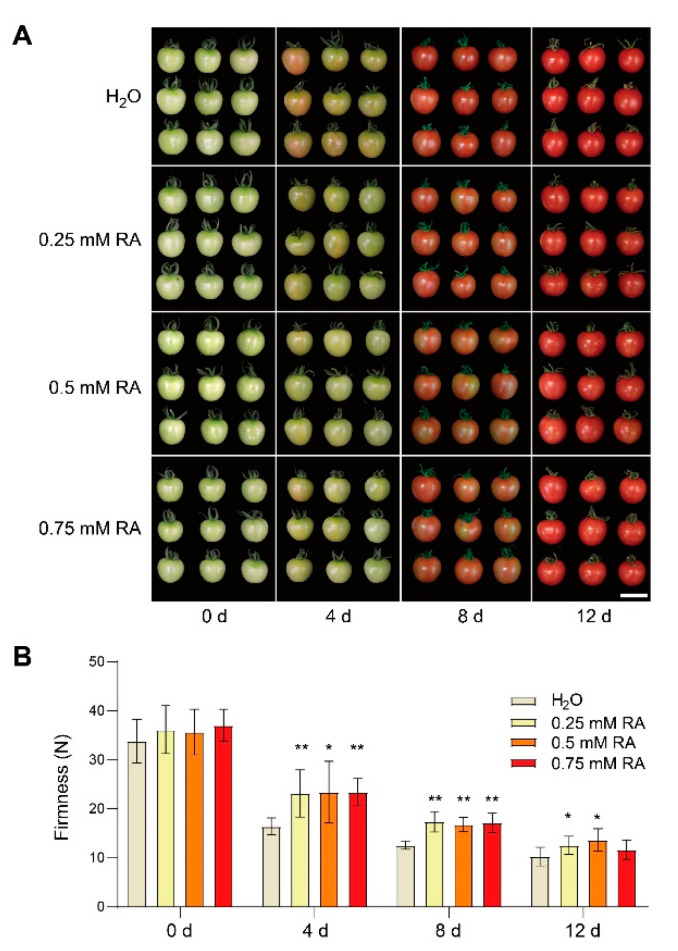
Rosmarinic acid (RA) postponed tomato fruit ripening. (**A**) The representative phenotype of tomato fruits at different days after RA treatment. Tomato fruits at the mature green stage were treated with 0.25, 0.5, and 0.75 mM RA, or H_2_O control. Bar = 2 cm. (**B**) Effect of RA on the fruit firmness at 0 d, 4 d, 8 d, 12 d after treatment. The data in (**B**) are presented as mean values ± SD; *n* = 9. Asterisks in (**B**) indicate statistically significant differences (* *p* ≤ 0.05, ** *p* ≤ 0.01) compared with control under the same time point, as determined by Student’s *t*-test.

Tomato fruits were collected at 0 d, 4 d, 8 d, and 12 d after soaking treatments with different RA concentrations or H_2_O as control. As illustrated in Figure 1A, 0.25 mM, 0.5 mM, and 0.75 mM RA soaking treatments inhibited tomato fruit color change during storage at 4 and 8 d, compared with the control treatment. In addition, RA treatment also maintained higher firmness than control during storage from 4 d to 12 d (Figure 1B). These results indicated that RA treatment retarded the color change and ripening of tomato fruits.

### 3.2. Effect of RA Treatment on Ripening-Induced Ethylene Production

**Figure 2:**
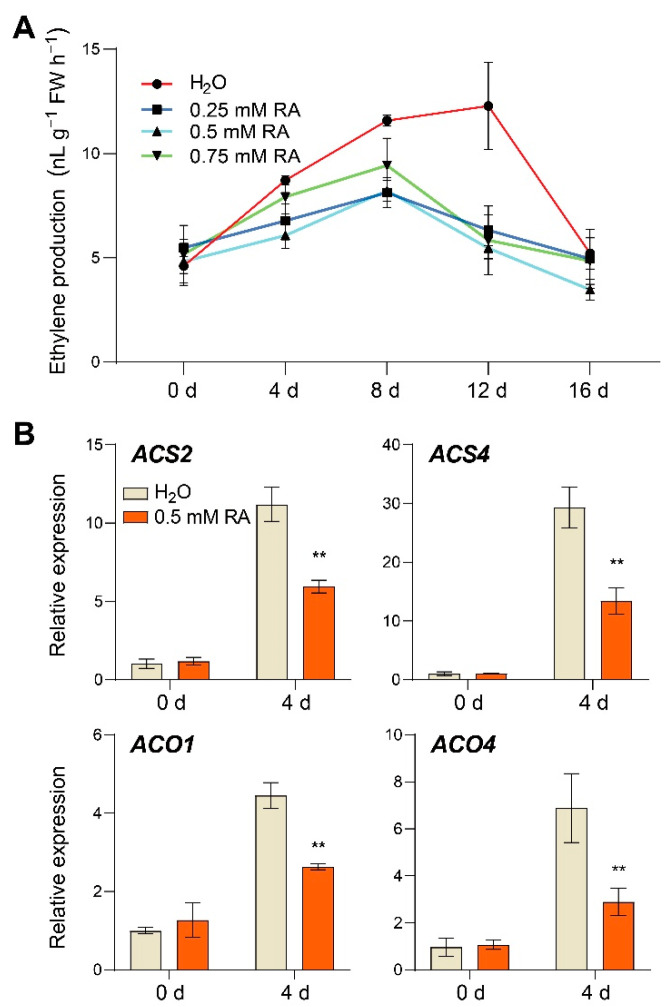
RA inhibited ripening-induced ethylene biosynthesis signaling. Effects of RA treatment on (**A**) ethylene production, and (**B**) transcript abundance of ethylene biosynthesis genes *ACS2*, *ACS4*, *ACO1*, and *ACO4*. The transcript abundance of each gene under control at 0 d was defined as 1. Tomato fruits at the mature green stage were treated with 0.25 mM, 0.5 mM, 0.75 mM RA, or H_2_O control. The fruit samples were collected at the indicated time points for ethylene content quantification and qRT-PCR analysis. The data are presented as mean values ± SD; *n* = 4 in (**A**), 3 in (**B**). Asterisks in (**B**) indicate statistically significant differences (** *p* ≤ 0.01) compared with control under the same time point, as determined by Student’s *t*-test.

Ethylene has long been implicated in ripening initiation, especially in climacteric fruits [5]. In order to address whether RA treatment affected ripening-induced ethylene biosynthesis, we quantified the ethylene production at different postharvest time points. As shown in Figure 2A, tomato fruits with control exhibited a typical ethylene burst after the onset of ripening, whereas RA treatments substantially inhibited ethylene production at 4 d, 8 d, and 12 d, especially with 0.5 mM RA. In line with the ethylene production, RA largely suppressed ripening-induced transcript abundance of ethylene biosynthesis genes, such as *ACS2*, *ACS4*, *ACO1*, and *ACO4* at 4 d after treatment (Figure 2B). Collectively, these results strongly suggest that RA inhibited ethylene production.

### 3.3. Effects of RA Treatment on Carotenoid Compositions

**Figure 3:**
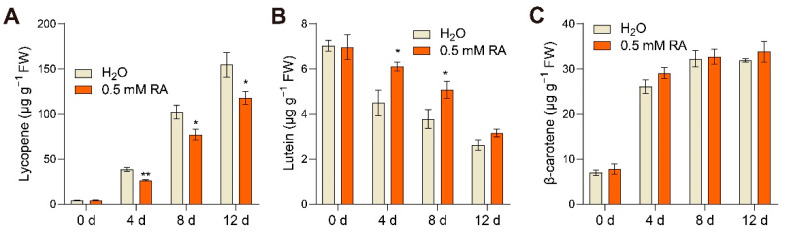
RA affected carotenoid biosynthesis during fruit ripening. Effects of RA treatment on (**A**) lycopene content, (**B**) lutein content, and (**C**) β-carotene content during fruit ripening. Tomato fruits at the mature green stage were treated with 0.5 mM RA, or H_2_O control. The fruit samples were collected at the indicated time points for carotenoid content quantification. The data are presented as mean values ± SD; *n* = 3. Asterisks indicate statistically significant differences (* *p* ≤ 0.05, ** *p* ≤ 0.01) compared with control under the same time point, as determined by Student’s *t*-test.

Carotenoid metabolism is a major fruit ripening trait, which primarily determines tomato fruit color. To explore whether RA treatment affected carotenoid biosynthesis during fruit ripening, we measured the accumulation of three typical carotenoids, including lycopene, lutein, and β-carotene. Time-course analysis revealed that the lycopene contents in tomato fruits gradually increased during fruit ripening, whereas ripening-induced lycopene accumulation was significantly inhibited at 4, 8, and 12 d after RA treatment compared to control (Figure 3A). In agreement with the deceased lycopene accumulation by RA treatment, the ripening-induced transcripts abundance of *Phytoene Desaturase* (*PDS*), *Zeta-Carotene Desaturase* (*ZDS*), and *Carotene Isomerase* (*CRTISO*), the genes involved in lycopene biosynthesis, was substantially suppressed at 4 d after RA treatment (Figure S1). In contrast, RA significantly prevented ripening-mediated lutein content reduction at both 4 and 8 d after RA (Figure 3B). Meanwhile, RA treatment did not affect β-carotene accumulation during fruit ripening (Figure 3C). Overall, RA differentially regulated carotenoid compositions and negatively affected lycopene accumulation, which was in accordance with the delayed color change in RA-treated fruits.

### 3.4. Effects of RA Treatment on Sugar and Organic Acid Accumulation

**Figure 4:**
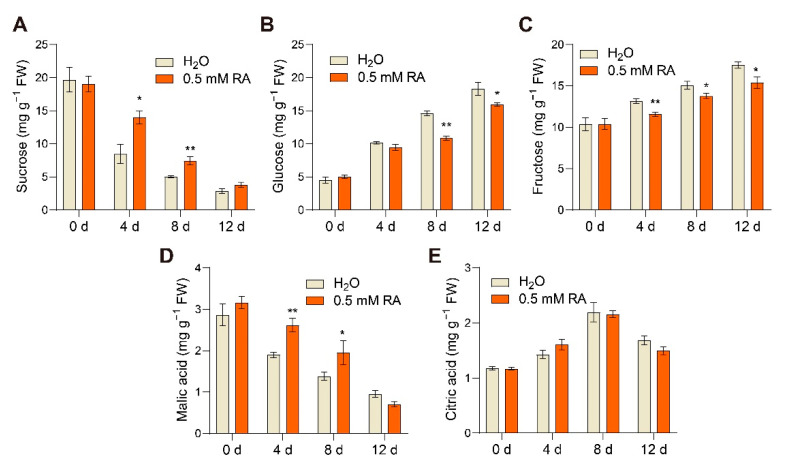
RA influenced sugar and organic acid accumulation during fruit ripening. Effects of RA treatment on (**A**) sucrose content, (**B**) glucose content, (**C**) fructose content, (**D**) malic acid content, and (**E**) citric acid content. Tomato fruits at the mature green stage were treated with 0.5 mM RA, or H_2_O control. The fruit samples were collected at the indicated time points for sugar and organic acid content quantification. The data are presented as mean values ± SD; *n* = 3. Asterisks indicate statistically significant differences (* *p* ≤ 0.05, ** *p* ≤ 0.01) compared with control under the same time point, as determined by Student’s *t*-test.

Tomato fruit ripening generally entails a dynamic metabolism of sugar and organic acid, which remarkably contribute to flavor [6]. To investigate the effects of RA treatment on fruit sugar accumulation, we determined the concentrations of sucrose, glucose, and fructose in tomato fruits with RA or control during fruit ripening. As illustrated in Figure 4A, RA largely prevented the decline in sucrose content during fruit ripening. Conversely, the ripening-induced monosaccharide accumulations of glucose and fructose were significantly inhibited (Figure 4B,C). Following the quantification assays, we also found that RA inhibited the decrease in malic acid, one of the primary acids in tomato fruits, during ripening (Figure 4D). However, RA did not affect critic acid content during ripening (Figure 4E). Taken together, these results indicated that RA treatment modestly influenced sugar and organic acid metabolism in fruits during ripening.

### 3.5. Effects of RA Treatment on Antioxidant System

**Figure 5:**
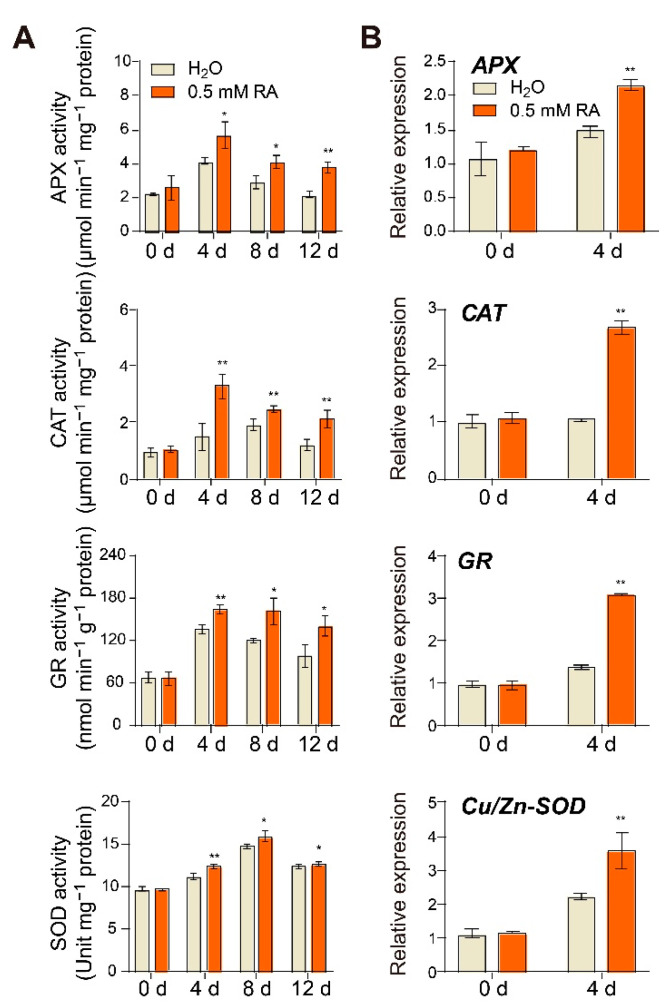
RA promoted transcript abundance and enzyme activity of antioxidant enzymes during fruit ripening. Effects of RA treatment on (**A**) antioxidant enzyme activity and (**B**) transcript abundance of antioxidant enzyme encoding genes. The transcript abundance of each gene under control at 0 d was defined as 1. APX, ascorbate peroxidase; CAT, catalase; GR, glutathione reductase; SOD, superoxide dismutase. Tomato fruits at the mature green stage were treated with 0.5 mM RA, or H_2_O control. The fruit samples were collected at the indicated time points for qRT-PCR and enzyme activity analysis. The data are presented as mean values ± SD; *n* = 4 in (**A**), 3 in (**B**). Asterisks indicate statistically significant differences (* *p* ≤ 0.05, ** *p* ≤ 0.01) compared with control under the same time point, as determined by Student’s *t*-test.

At early ripening stages, the increasing respiratory rates in tomato fruits are typically accompanied by a ROS burst [23]. The activation of antioxidant enzymes such as APX (ascorbate peroxidase), CAT (catalase), GR (glutathione reductase), and SOD (superoxide dismutase) could protect fruits against oxidative stress during fruit ripening and senescence [2]. To better understand the effects of RA treatment on the fruit antioxidant system, we analyzed the activity of antioxidant enzymes. As illustrated in Figure 5A, ripening-induced increase in the activity of APX, CAT, GR, and SOD was further promoted by RA treatment, and RA-mediated higher antioxidant enzyme activity could be still distinguished at 12 d after treatment. Consistent with the enzyme activity, the transcript abundance of genes encoding APX, CAT, POD, and SOD was also largely induced by RA treatment at 4 d of the early ripening stage (Figure 5B).

**Figure 6:**
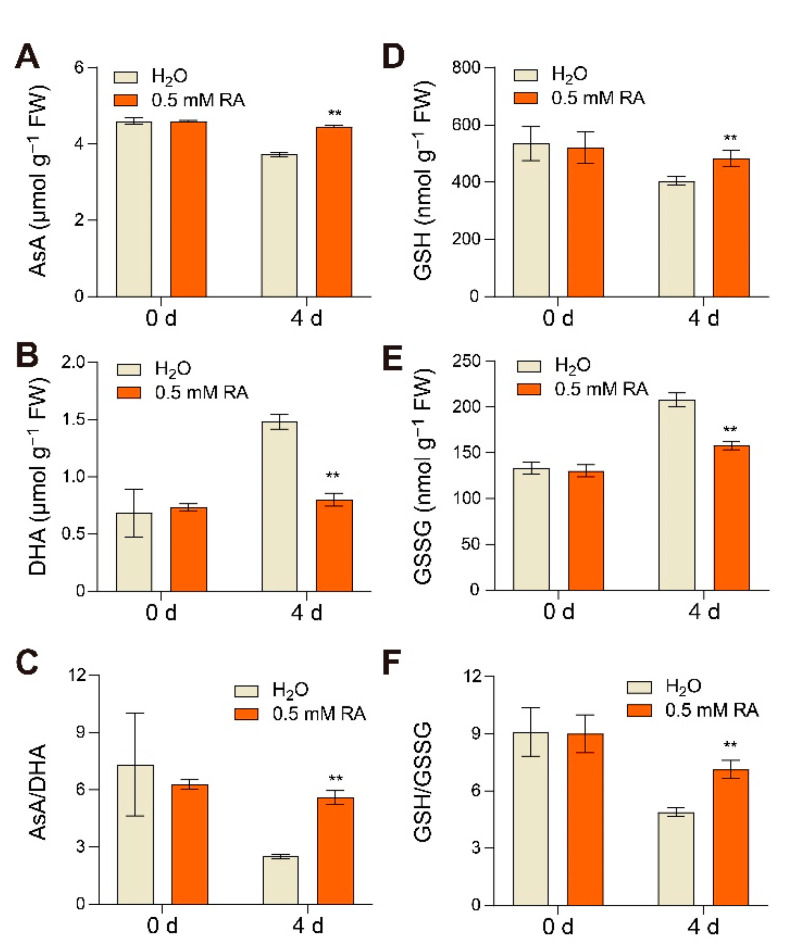
RA affected the redox status of antioxidant contents during fruit ripening. Effects of RA treatment on (**A**) reduced ascorbate (AsA) content, (**B**) dehydroascorbate (DHA) content, (**C**) redox status of ascorbate, (**D**) glutathione (GSH) content, (**E**) glutathione disulfide (GSSG) content, and (**F**) redox status of glutathione. Tomato fruits at the mature green stage were treated with 0.5 mM RA, or H_2_O control. The fruit samples were collected at the indicated time points for antioxidant quantification analysis. The data are presented as mean values ± SD; *n* = 4. Asterisks indicate statistically significant differences (** *p* ≤ 0.01) compared with control under the same time point, as determined by Student’s *t*-test.

Beside diverse antioxidant enzymes, antioxidant metabolites also play an indispensable role in the antioxidant system, among which ascorbate and glutathione act as the heart of the cytosol redox hub regulating redox homeostasis [24]. Generally, the changes in the ratios of reduced ascorbate to dehydroascorbate (AsA/DHA) and glutathione to glutathione disulfide (GSH/GSSG) reflect the alterations in redox status. The AsA content reduced and the DHA increased in H_2_O-treated fruits at 4 d of the early ripening stage, which led to a decline in the AsA/DHA ratio (Figure 6A–C). Notably, RA treatment was able to prevent the decline in AsA content, and inhibit the DHA accumulation, eventually keeping a higher level of the AsA/DHA ratio in fruits compared to that with control treatments (Figure 6A–C). Similarly, RA treatment also significantly induced the GSH content and substantially suppressed the GSSG content, protecting the GSH/GSSG ratio from further reduction during ripening (Figure 6D–F). These findings indicate that RA plays a positive role in strengthening the antioxidant system by activating antioxidant enzyme activity and regulating the redox status of antioxidant metabolite during tomato fruit ripening.

### 3.6. Effects of RA Treatment on Amino Acid Biosynthesis

**Figure 7:**
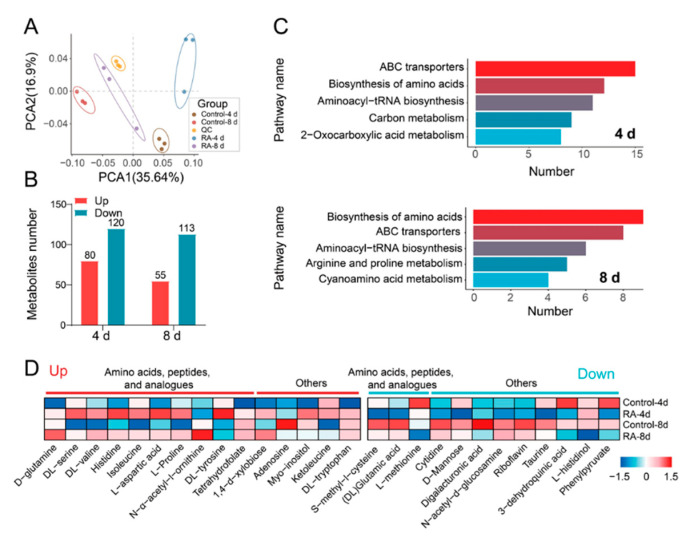
Effects of RA treatment on fruit metabolome. (**A**) PCA of metabolites as influenced by RA treatment at 4 and 8 d. (**B**) Number of metabolites increased or decreased by RA treatment at 4 and 8 d, respectively. (**C**) Top 5 enriched KEGG pathways based on RA-regulated differentially accumulated metabolites (DAMs) at 4 and 8 d, respectively. (**D**) Heatmap indicates the relative metabolite abundance of RA-regulated DAMs within the top 3 enriched KEGG pathways of ABC transporters, the biosynthesis of amino acids, and the aminoacyl-tRNA biosynthesis. The original metabolite abundance was subjected to data adjustment by normalization using an R package. Red and blue lines indicate the up- and down-regulated metabolites by RA treatment at 4 and 8 d, respectively. The color scale on the right indicates relative metabolites abundance.

During fruit ripening, a variety of metabolites are synthesized and degraded. To further clarify the global effects of RA on fruit ripening, we carried out an untargeted metabolomic analysis to recognize metabolite accumulation patterns of tomato fruits in response to RA treatment at 4 and 8 d. A total of 1328 metabolites were identified at two ripening stages (Table S2). The principal component analysis (PCA) revealed that the samples were divided into four groups, each associated with a treatment at a specific time point (Figure 7A). We identified 200 metabolites (80 upregulated and 120 downregulated in the RA-treated fruits compared with the H_2_O-treated fruits at 4 d), and 168 metabolites (55 upregulated and 113 downregulated in the RA-treated fruits compared with the H_2_O-treated fruits at 8 d) were differentially accumulated in positive or negative mode (VIP > 1, *p* ≤ 0.05), respectively (Figure 7B, Table S3). Strikingly, enrichment analysis of KEGG categories revealed that the differentially accumulated metabolites (DAMs) were mainly clustered into the pathways of ABC transports, the biosynthesis of amino acids, and the aminoacyl-tRNA biosynthesis both at 4 and 8 d (Figure 7C, Table S4). A close look into these enriched KEGG pathways showed that 10 out of 19 items in the ABC transports pathway, 13 out of 17 items in the biosynthesis of amino acids pathway, and 13 out of 13 items in the aminoacyl-tRNA biosynthesis pathway were associated with amino acids, peptides, and analogues (Table S5). According to the heat map of the accumulations of DAMs in these three pathways, the amino acids of D-glutamine, DL-serine, DL-valine, histidine, isoleucine, L-aspartic acid, L-proline, DL-tyrosine, and DL-tryptophan were enhanced by RA-treatment both at 4 and 8 d, whereas DL-glutamic acid and L-methionine were declined by exogenously applied RA (Figure 7D). Overall, the data revealed the specific roles of RA in tomato ripening and flavor formation potentially by regulating amino acid metabolism.

## 4. Discussion

A large number of signaling molecules derived from plants have been widely adopted over the decades to minimize the postharvest losses of fleshy fruits. Exogenous applications of melatonin, sucrose and several plant hormone molecules such as ethylene, abscisic acid, brassinolide, and methyl jasmonate accelerate postharvest tomato fruit ripening [9,25,26,27,28,29]. In contrast, nitric oxide, ethanol and other plant hormones like gibberellic acid (GA_3_) and salicylic acid have been demonstrated to delay tomato fruit ripening [2,30,31,32]. In this study, we found that RA, a natural polyphenol derived from the Rosmarinus plants, delayed fruit ripening and extended tomato fruit shelf life with less detrimental impacts on fruit quality. Thus, RA can be used as an alternative to minimize the postharvest losses of fleshy fruits.

Ethylene plays a predominant role in fruit ripening, which initiates and promotes the ripening-associated physiological process [5]. Generally, two systems of ethylene production are associated with climacteric fruits. System 1 is responsible for producing basal ethylene levels in all tissues, whereas system 2 is involved in ethylene production during fruit ripening, contributing to the autocatalytic regulation of climacteric ethylene [3]. In the present study, exogenous RA treatment appears to be a potent inhibitor of system 2 ethylene accumulation during fruit ripening (Figure 2A). Based on the genome-wide analysis, the presence of six *ACO* genes and 14 sequences corresponding to putative *ACS* genes in tomato was confirmed [3]. Among these ethylene biosynthesis gene families, *ACS2*, *ACS4*, and *ACO1* have been demonstrated to present the most striking ripening-regulated gene expression patterns at the breaker stage, whereas the *ACO4* transcript slightly increases throughout ripening [8]. Silencing these genes can slow or block tomato fruit ripening [33,34]. Here, our findings revealed the significant inhibitory effect of RA on the transcript abundance of these ripening-associated *ACS* and *ACO* genes (Figure 2B). Therefore, we inferred that RA treatment inhibited system 2 ethylene biosynthesis, thereby affecting the subsequent signaling pathway and delaying fruit ripening.

Tomato fruit ripening is accompanied by fruit color changes as the consequence of chlorophyll degradation and carotenoid accumulation. Generally, the red color of tomato fruits is largely determined by lycopene, which is the most abundant carotenoid hydrocarbon in tomato fruits [35]. Lycopene is biosynthesized via the central isoprenoid pathway starting from the formation of phytoene and next desaturation steps catalyzed by enzymes like PDS, ZDS, and CRTISO [36]. Here, we show that RA delays lycopene accumulation, as demonstrated by the lycopene contents and the transcript abundance of lycopene biosynthetic genes in tomato fruits treated with exogenous RA (Figure 3A and Figure S1). In addition, the color appearance of tomato fruits is also in agreement with this conclusion (Figure 1A). Previous studies show that inhibiting ethylene biosynthesis or reducing ethylene sensitivity significantly reduces lycopene biosynthesis during fruit ripening [18]. Considering the inhibitory impact of RA on ethylene biosynthesis, we speculate that the inhibition of lycopene in RA-treated fruits may be due to the effect of RA on the ethylene signaling. Further modifications of lycopene subsequently generate lutein in one branch and β-carotene in the other pathway [37]. Interestingly, unlike the inhibitory effect on lycopene accumulation, exogenous RA reduces the decline in lutein content, and has no significant impact on the increase in β-carotene during fruit ripening (Figure 3C,D). Hence, it remains to be studied why RA differentially regulates the accumulation of diverse carotenoid isoforms during fruit ripening.

In addition to carotenoid accumulation and metabolism, changes of flavor metabolites such as sugars, organic acids, and amino acids are strongly associated with tomato organoleptic quality [2]. Soluble sugar sucrose and its hydrolysis products fructose and glucose are positively correlated with a sweet flavor. Generally, green tomato fruits contain higher sucrose contents than the red mature ones, because ripening induces sucrose hydrolysis accompanied with increases in glucose and fructose contents [38]. In the present study, exogenous RA application significantly inhibited sucrose degradation and monosaccharide accumulation during ripening (Figure 4A–C). Similarly, the other fruit ripening inhibitor nitric oxide suppresses sucrose content but promotes fructose and glucose accumulation at the breaker stage of ripening [2]. In contrast, sucrose degradation is accelerated by exogenous application of methyl jasmonate or ethylene, which both positively regulate fruit ripening [38,39]. Here, we also found the inhibitory effect of RA on malic acid degradation, which was similar to that in sucrose (Figure 4D). This is in agreement with a previous study, which showed that suppression of the decline rate of malic acid by exogenous 1-naphthaleneacetic acid (NAA) application was also associated with the delayed ripening in grape berry [40]. According to the metabolome result, multiple DAMs identified in RA-treated fruits were classified into KEGG pathways related to amino acids, peptides, and analogues, of which 10 DAMs were up-regulated, and 3 DAMs were downregulated by exogenous RA treatment (Figure 7). It was noted that these RA-enhanced amino acids, including aspartic acid, serine, tyrosine, also inhibited the postharvest senescence in broccoli by suppressing ethylene production and respiration rate when exogenously applied [41]. Interestingly, aspartic acid, regarded as a major contributor to fruit flavor [42], was positively modulated by RA. Moreover, proline was also found enriched in RA-treated fruits, potentially associated with its important role in cellular ROS balance [43].

Fruit ripening has been characterized as an oxidative event, which requires a regulation of ROS turnover to sustain redox homeostasis [44]. Therefore, the antioxidant system plays a pivotal role in the ripening process, wherein enhanced antioxidant-enzyme activity contributes to ROS scavenging and in turn delays fruit ripening and senescence [45]. Our assays also confirm that the enzyme activity of APX, CAT, GR and SOD was induced by ripening, and their transcripts and activities were further promoted by exogenous RA treatment (Figure 5). In accordance with our results, previous studies have shown that RA caused a remarkably increase in the activity of CAT, SOD, and glutathione peroxidase leading to a decrease in lipid peroxidation level in the aging mice [46]. Notably, RA itself can directly scavenge free radicals, chelate pro-oxidant ions, and so is regarded to be the strongest antioxidant of all hydroxycinnamic acid derivatives [46]. Besides the increases in antioxidant enzyme activity, RA also regulates the redox status of ascorbate and glutathione, which hold both high levels of AsA/DHA and GSH/GSSG (Figure 6). Our finding is also supported by a previous study that the adventitious effect of RA on redox homeostasis relied on the increased ratio of GSH/GSSG in rats [47]. Both ascorbate and glutathione are abundant and stable antioxidants, not only playing irreplaceable roles in plant growth and development, but also providing beneficial nutraceuticals to human beings [24,48].

## 5. Conclusions

Our study highlighted an important efficiency of RA in fruit postharvest storage, wherein exogenous RA treatment postponed tomato fruit ripening. Evidence presented here showed that RA application caused a substantial decrease in ripening-associated ethylene production, impaired lycopene accumulation. and inhibited degradations of sucrose and malic acid during tomato fruit ripening. Besides, RA-mediated delayed ripening in tomato fruits may be attributed to the higher accumulation of multiple amino acids, and the balance of cellular redox regulated by effective antioxidant enzymes and antioxidants. To sum up, this study provides a promising strategy that can be potentially utilized to adjust fruit postharvest longevity.

## Data Availability

The data presented in this study are available in article and supplementary material.

## Supplementary Materials

The following are available online at https://www.mdpi.com/article/10.3390/antiox10111821/s1, Figure S1: Effects of RA treatment on transcript abundance of genes involved in carotenoid biosynthesis. Table S1: Primer sequences used for qRT-PCR; Table S2: List and characteristics of the metabolites identified and quantified at 4 and 8 d after soaking treatments with RA; Table S3: List and characteristics of the identified different metabolites at 4 and 8 d after soaking treatments with RA; Table S4: Enriched KEGG pathway at 4 and 8 d after soaking treatments with RA; Table S5: Metabolites in ABC transporters, biosynthesis of Amino acid and Aminoacyl-tRNA biosynthesis KEGG pathway at 4 and 8 d after soaking treatments with RA.
