# Supplementary material for: Rosmarinic Acid Delays Tomato Fruit Ripening by Regulating Ripening-Associated Traits
Source: Antioxidants (Basel). 2021 Nov 17;10(11):1821. doi: 10.3390/antiox10111821 (PMC8614985; doi:10.3390/antiox10111821)
Supplement: Supplementary file 1 [file antioxidants-10-01821-s001.zip › Supplemental__Figures.pdf]

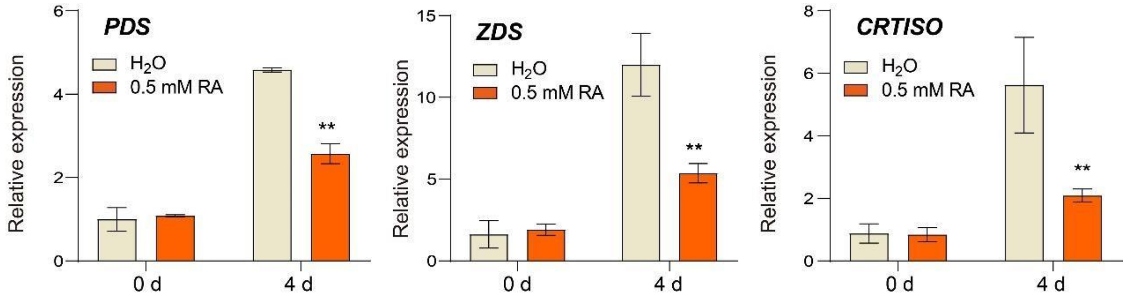

Figure S1. Effects of RA treatment on transcript abundance of genes involved in carotenoid biosynthesis. The transcript abundance of *Phytoene Desaturase* (*PDS*), *Zeta-Carotene Desaturase* (*ZDS*), and *Carotene Isomerase* (*CRTISO*) each gene under control treatment at 0 d was defined as 1. The fruit samples were collected at the indicated time points for and qRT-PCR analysis. The data are presented as mean values ± SD; $n = 3$. Asterisks indicate statistically significant differences (**$P \leq 0.01$) compared with control treatment under the same time point, as determined by Student's *t*-test
